# Demographics, clinical characteristics, and outcomes in hospitalized patients during six waves of COVID‑19 in Northern Iran: a large cohort study

**DOI:** 10.1038/s41598-023-50139-8

**Published:** 2023-12-18

**Authors:** Hoda Shirafkan, Farzin Sadeghi, Mehrdad Halaji, Rabeae Rahmani, Yousef Yahyapour

**Affiliations:** 1 Social Determinants of Health Research Center, Health Research Institute, Babol University of Medical Science, Babol, Iran; 2 Cellular and Molecular Biology Research Center, Health Research Institute, Babol University of Medical Sciences, Babol, Iran; 3 Infectious Diseases and Tropical Medicine Research Center, Health Research Institute, Babol University of Medical Sciences, Babol, Iran; 4 Biomedical and Microbial Advanced Technologies Research Center, Health Research Institute, Babol University of Medical Sciences, Babol, Iran; 5 Department of Medical Microbiology and Biotechnology, Faculty of Medicine, Babol University of Medical Sciences, Babol, Iran; 6 Cellular and Molecular Biology, Education of Amol Teacher, Amol, Iran

**Keywords:** Virology, SARS-CoV-2, Viral epidemiology

## Abstract

Since the first report of coronavirus disease 2019 (COVID-19) in Iran, our country has experienced several waves of severe acute respiratory syndrome coronavirus 2 (SARS-CoV-2) infection. Northern Iran was one of the most affected regions of the country by COVID-19. In the current study, the demographic and clinical characteristics and outcomes of hospitalized patients were determined over a 2-year period (during six waves of SARS-CoV-2). This is a large cohort study investigating hospitalized patients with suspected and probable, and confirmed SARS-CoV-2 infection in Babol district, northern Iran, during the two years of COVID-19. The study population included patients admitted to four hospitals affiliated with Babol University of Medical Sciences between March 7, 2020 (start of the first wave) and March 20, 2022 (end of the sixth wave). Epidemiological and demographic characteristics, real-time PCR, cycle thresholds, clinical data and outcomes of COVID-19 were analyzed in 24,287 hospitalized patients. A total of 24,287 hospitalized patients were included in the study: 13,250 (46.6%) patients were suspected of having COVID-19, 11037(45.4%) were confirmed COVID-19 cases. The mean age of confirmed COVID-19 patients was 54.5 ± 18.9 years and 5961 (54%) were female. The median length of hospitalization for COVID-19 survivors and non-survivors was 5 (interquartile range [IQR] 4-8) and 7 (IQR 3-15) days, respectively. Of the patients with confirmed COVID-19, 714 (6.5%) died during hospitalization. In addition, the mortality rate from the first to the sixth wave was 22.9%, 8.1%, 9.9%, 6.8%, 2.7% and 3.5% in confirmed COVID-19 patients. The patients in the fifth wave were significantly younger than the others (mean age and SD of 51.1 ± 17.4 versus 59.2 ± 16.9, 54.7 ± 19.9, 58.4 ± 17.9, 53.5 ± 16.8 and 58.5 ± 25.1 years; p<0.001). The highest in-hospital mortality rate was 22.9% (126/551) in the first wave and the lowest in the fifth wave was 2.7% (96/3573) of cases. In conclusion, in the present study, the in-hospital mortality rate was 6.5% and more than half of the deceased patients were ≥65 years old. Male gender, advanced age and comorbidities significantly increased the mortality rate. The patients in the fifth wave were significantly younger than those in the other waves, and the lowest mortality rate and intensive care unit admission were also observed in the fifth wave.

## Introduction

The severe acute respiratory syndrome coronavirus 2 (SARS-CoV-2) as a very contagious and pathogenic coronavirus emerged in late 2019 and caused a pandemic respiratory disease known as “Coronavirus Disease 2019” (COVID-19)^1^. The first cases of COVID-19 in Iran were described in Qom province on February 19, 2019^2^. After that, the disease spread rapidly throughout the country, especially in the northern provinces, which are attractive to tourists. The increasing number of patients with COVID-19 has led to more and more patients having to be admitted to hospital and the intensive care unit (ICU). The difference in mortality rates between countries may be caused by attention to emergencies in terms of hospital beds, trained healthcare workers, protective equipment and the use of different medications^3^.

As of June 14, 2023, 767,984,989 confirmed cases of COVID-19 with 6,943,390 deaths have been reported to the World Health Organization (WHO) worldwide, and in Iran, there were 7,612,001 confirmed cases of COVID-19 from January 3, 2020 to June 14, 2023, resulting in 146,273 deaths.

Some studies have considered the risk factors and mortality rate of SARS-CoV-2 infection^4–7^, but there is no conclusive data from Iran as one of the major epicenters of COVID-19.

COVID-19 vaccines have helped contain the disease, but the emergence of new Variants of Concern (VOCs) that can evade the immune response has led to an increase in the disease burden^8,9^. During the pandemic, different variants of SARS-CoV-2 were discovered, some of which have spread worldwide, while others have quickly disappeared. The new variants of SARS-CoV-2 classified as VOCs by the WHO in 2021^10^. These variants lead to multiple waves of COVID-19 occurring in most countries.

Several variants of SARS-CoV-2 have emerged during the pandemic, some of which have spread worldwide, while others have quickly disappeared^11^. In Iran, the outbreak of the SARS-CoV-2 pandemic occurred in six different waves until October 2021. The first wave of infection was observed from February to May 2020, (related to the original Wuhan strain), followed by the second wave from June to September 2020 (related to the original Wuhan strain). Then, the third wave (related to the spike mutation D614G) happened from the end of November to the beginning of March 2020. Moreover, the fourth wave (related to the Alpha variant) was launched at the end of March until the end of May 2021, and the fifth wave (related to the Delta variant) started at the end of June and lasted until December 2021, while the sixth wave (related to the Omicron variant) was detected from January to March 20, 2022^12–14^.

The aim of the current two-year retrospective cohort study was to investigate the ICU admission and in-hospital mortality of COVID-19 patients over two years in the March 7, 2020 and March 20, 2022 from the beginning of the first to the end of the sixth wave in Iran. In addition, this study was conducted to determine the demographic data, clinical characteristics, relative viral load and outcomes of COVID-19 patients admitted to the hospitals of Babol district, Mazandaran province, and northern Iran in six waves over a two-year period.

## Methods

### Study design

**Figure 1:**
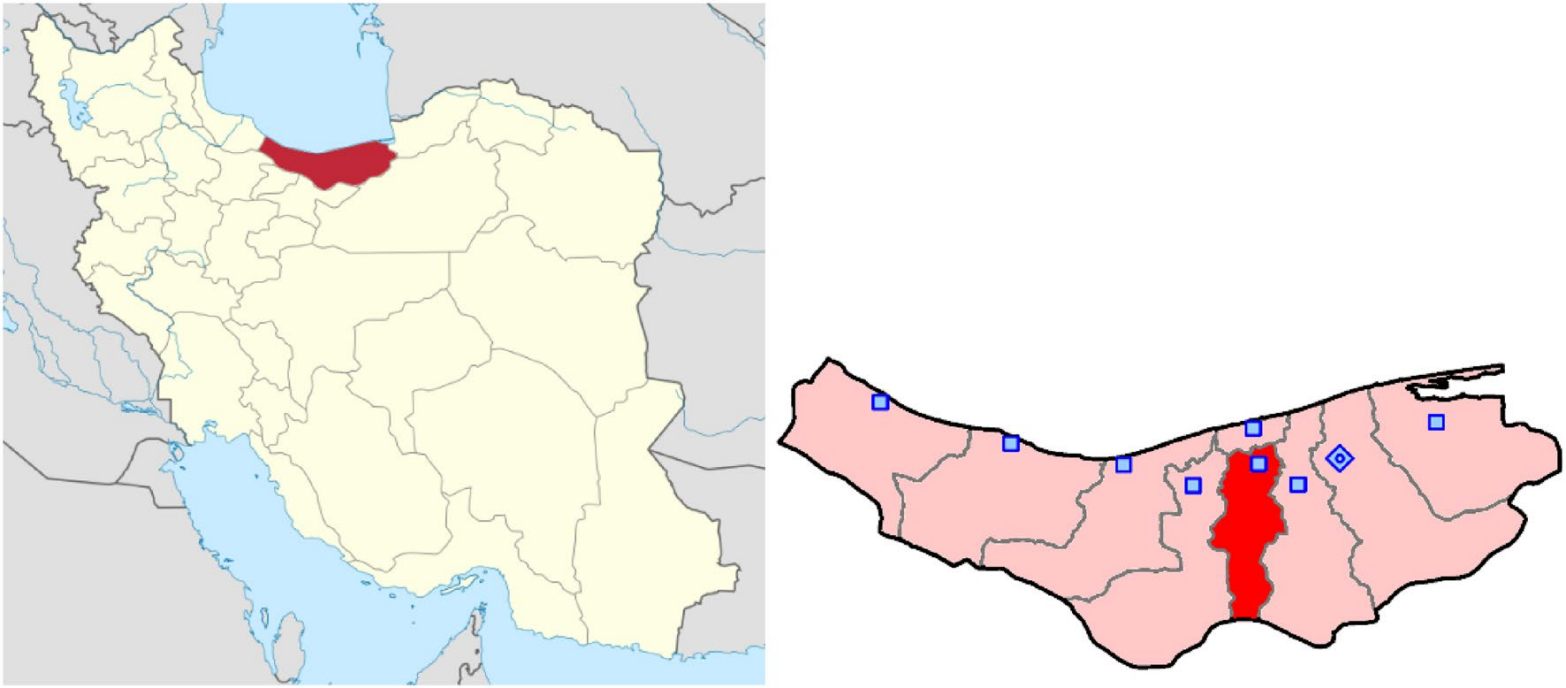
Map of Iran showing the location of the district of Babol in the province of Mazandaran in northern Iran (Babol district is shown in Highlight, From Wikipedia, the free encyclopedia, https://en.wikipedia.org/wiki/File:Iran_location_map.svg).

The province of Mazandaran is one of the 31 provinces of Iran and is located in the north of the country near the Caspian Sea. According to the 2021 census, it is the 7th most populous province in Iran with 3,375,900 inhabitants. Mazandaran Province is one of the most populous and densely populated regions in Iran with 22 counties. Babol district is the most populous district in Mazandaran province with 546,800 inhabitants. Babol is the center of medical care in Mazandaran province and receives many patients from neighboring counties or provinces every year (Fig. 1).

All patients hospitalized between March 7, 2020 and March 20, 2022 suspected or laboratory-confirmed COVID-19 in four university hospitals in Babol district were included in this retrospective multicenter large cohort study.

All hospitalized patients were followed up until discharge or death. The inclusion criteria were hospitalizations with suspected, probable or confirmed diagnosis of COVID-19 during the study.

Subjects without a real-time reverse transcription polymerase chain reaction (rRT-PCR) test result and duplicate data sets based on name, ID number, and gender were excluded from the study.

### Data collection and sources

The studied population was patients of all age groups suspected of having COVID-19. The criteria for the definition of suspected COVID-19 cases corresponded to those of the WHO. SARS-CoV-2 infection was confirmed by rRT-PCR at the Molecular Diagnostic Reference Laboratory for SARS-CoV-2 affiliated with Babol University of Medical Sciences. For each patient, demographic variables such as age and gender, self-reported history of underlying disease (including cardiovascular disease (CVD), diabetes, hypertension, kidney disease (KD), cancer, brain and neurological disease (BND), pregnancy, etc.), as well as rRT-PCR result, ICU admission and in-hospital outcome (discharge or death) were recorded.

### Real-time RT-PCR (rRT-PCR) and relative viral load

Viral nucleic acid extraction and rRT-PCR for SARS-CoV-2 detection were performed as described in previous studies^15,16^. The cycle threshold (Ct) values obtained from rRT-PCR were used to determine the relative viral loads of SARS-CoV-2 positive samples. Based on the Ct values, the patients were classified into three groups: with Ct values between 9-20, 21-30, and 31-40^16^.

### COVID-19 waves

Over a period of 2 years, from March 7, 2020 to March 20, 2022, Mazandaran Province and Babol district experienced six waves of COVID-19. The first wave started on March 7, 2020 until May 16, 2020 (about 71 days); the second wave started on June 10, 2020 until September 5, 2020 (about 88 days); the third wave started on November 25, 2020 until March 5, 2021 (about 101 days); the fourth wave started on March 25, 2021 until May 31, 2021 (about 68 days); the fifth wave started on June 26, 2021 until December 1, 2021 (about 159 days); and the sixth wave started on January 17, 2022 until March 20, 2022 (about 63 days). In addition, there were approximately 208 days between waves (as a null wave) over a period of 2 years.

### Statistical analysis

SPSS 22 was used to analyze the data. Categorical variables were summarized as frequencies and percentages. Quantitative variables were presented as mean ± standard deviation (SD), median and IQR. Chi-square, generalized Fisher exact tests and Mann–Whitney *U*-test were used for comparison between groups and categories, respectively. In addition, the median test and Kruskal-Wallis test were used for median and mean age values. In addition, a multivariable Cox regression was performed to evaluate the factors influencing patient survival. A stepwise backward test (likelihood ratio) was used to estimate the coefficients of the Cox model (hazard ratio (HR)).

### Patient and public involvement

Patients and/or the public were not involved in the design, planning, management, and conduct of the research.

### Ethics approval and consent to participate

The present study was conducted in accordance with the guidelines of the Declaration of Helsinki and all procedures involving human subjects were approved by the Ethics Committee of Babol University of Medical Sciences (No. IR.MUBABOL.HRI.REC.1401.271). All participating patients gave written informed consent before participating in the study.

## Results

Over a period of two years, a total of 24,287 patients with suspected COVID-19 and a mean age of 52.4 ± 23.9 years (median: 56, IQR: 38-70) were enrolled in the study (46.7% female). Moreover, 11,037 laboratory-confirmed SAR-CoV-2 patients with a mean age of 54.5 ± 18.9 years (median, IQR: 56, 42-68) were included in the present study (54% female). Of all confirmed SARS-CoV-2 cases, 7197 (65.2%) were in the 18–64 age group. Tables 1 and 2 show the demographic and clinical characteristics of 24,287 and 11,037 suspected and confirmed COVID-19 patients included in this study, respectively.

**Table 1:** Characteristics and outcomes in 24,287 patients hospitalized with suspected COVID-19 by rRT-PCR status in Babol district in six waves (from March 7, 2020 to March 20, 2022).

Variable	Total, N (%)	First wave, N (%)	Second wave, N (%)	Third wave, N (%)	Fourth wave, N (%)	Fifth wave, N (%)	Sixth wave, N (%)	Null wave^1^, N (%)	*p*-value
Overall	24287	1598	2771	2563	2519	5231	2143	7462	–
Median age, yrs, (IQR)	56 (38–70)	60 (45–71)	57 (39–71)	58 (40–71)	56 (39–70)	52 (37–65)	62 (38–76)	58 (37–72)	<0.001
Mean ± SD, age, yrs	52.4±23.9	56.6±20.8	53.4±23.1	53.5±24.2	53.6±21.8	49.5±21.7	54.3±27.5	52.5±25.5	<0.001
Age group, yrs									
<18	2731 (11.3)	89 (5.6)	270 (9.7)	304 (11.9)	185 (7.4)	522 (10)	349 (16.4)	1012 (13.7)	<0.001
18–49	6547 (27.1)	415 (26)	767 (27.7)	633 (24.7)	781 (31)	1803 (34.6)	361 (16.9)	1787 (24.1)	
50–64	6359 (26.3)	469 (29.3)	763 (27.5)	659 (25.7)	686 (27.3)	1557 (29.9)	463 (21.7)	6359 (26.3)	
65–79	5644 (23.3)	419 (26.2)	621 (22.4)	628 (24.5)	588 (23.4)	977 (18.7)	573 (26.9)	1838 (24.8)	
≥80	2918 (12.1)	206 (12.9)	350 (12.6)	337 (13.2)	256 (11)	353 (6.8)	388 (18.2)	1008 (13.6)	
Sex									
Male	11924 (49.1)	851 (53.3)	1433 (51.7)	1309 (51.1)	1188 (47.2)	2392 (45.7)	1005 (46.9)	3746 (50.2)	<0.001
Female	12363 (50.9)	747 (46.7)	1338 (48.3)	1254 (48.9)	1331 (52.8)	2839 (54.3)	1138 (53.1)	3716 (49.8)	
rRT-PCR status									
Positive	11037 (45.4)	551 (34.5)	1526 (55.1)	1552 (60.6)	1402 (55.7)	3573 (68.3)	835 (39)	1598 (21.4)	<0.001
Negative	13250 (44.6)	1047 (65.5)	1245 (44.9)	1011 (39.4)	1117 (4.3)	1657 (31.7)	1308 (61)	5864 (78.6)	
Underlying diseases									
CVD^2^	6782 (27.9)	489 (30.6)	952 (34.4)	851 (33.2)	652 (25.9)	857 (16.4)	635 (29.6)	2346 (31.4)	<0.001
Diabetes	5010 (20.6)	441 (27.6)	733 (26.5)	653 (25.5)	433 (17.2)	751 (14.4)	397 (18.5)	1602 (21.5)	<0.001
KD^3^	1026 (4.2)	97 (6.1)	180 (6.5)	116 (4.5)	74 (2.9)	103 (2)	80 (3.7)	376 (5)	<0.001
Hypertension	2542 (10.5)	124 (7.8)	386 (13.9)	258 (10.1)	195 (7.7)	372 (7.1)	239 (11.2)	968 (13)	<0.001
Malignancies	1313 (5.4)	64 (4)	153 (5.5)	82 (6.2)	123 (4.9)	160 (3.1)	122 (5.7)	609 (8.2)	<0.001
BND^4^	1453 (6)	95 (5.9)	180 (6.5)	151 (5.9)	132 (5.2)	179 (3.4)	160 (7.5)	556 (7.5)	<0.001
RD^5^	861 (3.5)	78 (4.9)	113 (4.1)	93 (3.6)	82 (3.3)	100 (1.9)	73 (3.4)	322 (4.3)	<0.001
GID^6^	100 (0.4)	18 (1.1)	32 (1.2)	6 (0.2)	6 (0.2)	1 (0.02)	1 (0.05)	36 (0.5)	<0.001
LD^7^	204 (0.8)	18 (1.1)	30 (1.1)	16 (0.6)	18 (0.7)	30 (0.6)	11 (0.5)	81 (1.1)	0.006
HBD^8^	201 (0.8)	28 (1.8)	35 (1.3)	20 (0.8)	18 (0.7)	14 (0.3)	13 (0.6)	73 (1)	<0.001
Pregnancy	281 (1.2)	11 (0.7)	30 (1.1)	14 (0.5)	19 (0.8)	73 (1.4)	30 (1.4)	104 (1.4)	0.001
Others^9^	231 (1)	16 (1)	52 (1.9)	15 (0.6)	31 (1.2)	31 (0.6)	21 (1)	65 (0.9)	<0.001
Comorbidities									
No comorbidities	11209 (46.2)	673 (42.1)	1055 (38.1)	1043 (40.7)	1219 (48.4)	3346 (64)	967 (45.1)	2906 (38.9)	<0.001
One	8061 (33.2)	544 (34)	917 (33.1)	935 (36.5)	917 (36.4)	1258 (24)	725 (33.8)	2765 (36.1)	
≥ Two	5017 (20.7)	381 (23.8)	799 (28.8)	585 (22.8)	383 (15.2)	627 (12)	451 (21)	1791 (24)	
Hospitalized outcome									
Length of stay, days, median (IQR)	5 (4–8)	6 (3–10)	6 (3–9)	5 (4–8)	5 (4–8)	5 (4–7)	5 (3–8)	5 (4–8)	<0.001
Length of stay, days, mean ± SD	7.5±8.9	8.6±11.1	8.2±9.9	7.3±7.1	7.5±11.3	7.1±8.6	6.6±6.1	7.6±8.5	<0.001
ICU admission	891 (3.7)	83 (5.2)	108 (3.9)	83 (3.2)	97 (3.9)	176 (3.4)	75 (3.5)	269 (3.6)	0.028
In-hospital death	1236 (5.1)	211 (13.2)	186 (6.7)	234 (9.1)	167 (6.6)	139 (2.7)	43 (2)	256 (3.4)	<0.001

^1^Null wave: related to between waves of SARS-CoV-2 infections (about 208 days), ^2^*CVD* cardiovascular diseases, ^3^*KD* kidney diseases, ^4^*BND* brain & neurologic disorders, ^5^*RD* respiratory diseases, ^6^*GID* gastrointestinal diseases, ^7^*LD* liver diseases, ^8^*HBD* hematopoietic & blood disorders and ^9^Others: diseases of immunodeficiency, lupus, special diseases, and thyroid.

**Table 2:** Characteristics and outcomes in 11,037 confirmed COVID-19 hospitalized patients in a period when the SARS-CoV-2 variant was prevalent in Babol district, northern Iran, in six waves (from March 7, 2020 to March 20, 2022).

Variable	Total, N (%)	First wave, N (%)	Second wave, N (%)	Third wave, N (%)	Fourth wave, N (%)	Fifth wave, N (%)	Sixth wave, N (%)	Null wave^1^, N (%)	*p*-value
Overall	11037	551	1526	1552	1402	3573	835	1598	–
Median age, yrs, (IQR)	56 (42–68)	60 (47–72)	57 (41–69)	59 (47–71)	53 (40–65)	52 (40–63)	65 (44–78)	57 (42–69)	<0.001
Mean ± SD, age, yrs	54.5±18.9	59.2±16.9	54.7±19.9	58.4±17.9	53.5±16.8	51.1±17.4	58.5±25.1	55.4±19.5	<0.001
Age group, yrs									
<18	391 (3.5)	3 (0.5)	70 (4.6)	30 (1.9)	18 (1.3)	121 (3.4)	84 (10.1)	65 (4.1)	<0.001
18–49	3735 (33.9)	158 (28.7)	486 (31.8)	433 (27.9)	568 (40.4)	1445 (40.5)	160 (19.2)	486 (3.4)	
50–64	3462 (31.4)	175 (31.8)	480 (31.5)	484 (31.2)	444 (31.7)	1215 (34)	166 (19.9)	498 (31.2)	
65–79	2143 (21.9)	142 (25.8)	323 (21.2)	405 (26.1)	276 (19.7)	642 (18)	246 (29.5)	379 (23.7)	
≥80	1032 (9.4)	73 (13.2)	167 (10.9)	200 (12.9)	97 (6.9)	149 (4.2)	177 (21.2)	169 (10.6)	
Sex									
Male	5076 (46)	315 (57.2)	745 (48.8)	737 (47.5)	614 (43.8)	1533 (42.9)	377 (45.1)	755 (47.2)	<0.001
Female	5961 (54)	236 (42.8)	781 (51.2)	815 (52.5)	788 (56.2)	2040 (57.1)	458 (54.9)	843 (52.8)	
Mean Ct (relative viral load)*									
9–20	2140 (19.5)	18 (3.3)	214 (14)	112 (7.2)	444 (32.8)	919 (25.9)	226 (27)	207 (13)	<0.001
21–30	6172 (56.3)	226 (41)	748 (49)	884 (56.9)	742 (54.8)	2207 (62)	502 (60)	863 (54.3)	
31–40	2653 (24.2)	307 (55.7)	565 (37)	557 (35.9)	169 (12.5)	431 (12.1)	108 (12.9)	519 (32.7)	
Underlying diseases									
CVD^2^	2842 (24.8)	132 (24.1)	517 (33.9)	581 (37.4)	298 (21.3)	484 (13.5)	243 (29.1)	486 (30.3)	<0.001
Diabetes	2356 (21.3)	142 (25.8)	448 (29.3)	469 (30.2)	236 (16.8)	520 (14.5)	174 (20.8)	367 (22.9)	<0.001
KD^3^	313 (2.8)	18 (3.3)	71 (4.6)	44 (2.8)	33 (2.4)	53 (1.5)	34 (4.1)	60 (3.7)	<0.001
Hypertension	1121 (10.1)	33 (6)	254 (16.6)	167 (10.8)	101 (7.2)	232 (6.5)	93 (11.1)	241 (15)	<0.001
Malignancies	259 (2.3)	8 (1.5)	48 (3.1)	31 (2)	29 (2.1)	47 (1.3)	40 (4.8)	56 (3.5)	<0.001
BND^4^	463 (4.2)	10 (1.8)	78 (5.1)	84 (5.4)	48 (3.4)	84 (2.4)	75 (9)	84 (5.2)	<0.001
RD^5^	309 (2.8)	14 (2.5)	61 (4)	58 (3.7)	49 (3.9)	54 (1.5)	29 (3.5)	44 (2.7)	<0.001
GID^6^	17 (0.2)	3 (0.5)	8 (0.5)	2 (0.1)	2 (0.1)	1 (0.03)	1 (0.1)	0	<0.001
LD^7^	69 (0.6)	2 (0.4)	14 (0.9)	8 (0.5)	11 (0.8)	10 (0.3)	7 (0.8)	17 (1.1)	0.014
HBD^8^	53 (0.5)	5 (0.9)	10 (0.7)	9 (0.6)	10 (0.7)	9 (0.3)	3 (0.4)	7 (0.4)	0.108
Pregnancy	136 (1.2)	4 (0.7)	21 (1.4)	12 (0.8)	8 (0.6)	54 (1.5)	25 (3)	12 (0.7)	<0.001
Others^9^	99 (0.9)	8 (1.5)	30 (2)	8 (0.5)	21 (1.5)	13 (0.4)	11 (1.3)	8 (0.5)	<0.001
Comorbidities									
No comorbidities	5757 (52.1)	288 (52.3)	604 (39.6)	591 (38.1)	767 (54.7)	2459 (68.8)	344 (41.1)	704 (43.9)	<0.001
One	3239 (29.3)	172 (31.2)	463 (30.3)	558 (35.9)	455 (32.5)	749 (21)	301 (36)	541 (33.8)	
≥ Two	2049 (18.6)	91 (16.5)	460 (30.1)	404 (26)	180 (12.8)	366 (10.2)	191 (22.8)	357 (22.3)	
Hospitalized outcome									
Length of stay, days, median (IQR)	5 (4–8)	6 (3–9)	6 (3–9)	5 (4–8)	5 (4–7)	5 (4–7)	5 (3–7)	5 (4–8)	<0.001
Length of stay, days, mean ± SD	7.1±8.1	7.7±7.2	8.2±9.8	6.9±6.3	6.6±9.4	6.7±7.5	6.3±5.7	7.7±9.2	<0.001
ICU admission	485 (4.4)	39 (7.1)	57 (3.7)	49 (3.2)	62 (4.4)	138 (3.9)	42 (5)	98 (6.1)	<0.001
In-hospital death	714 (6.5)	126 (22.9)	124 (8.1)	154 (9.9)	95 (6.8)	96 (2.7)	29 (3.5)	90 (5.6)	<0.001

^1^Null wave related to between waves of SARS-CoV-2 infections (about 208 days), ^2^*CVD* cardiovascular diseases, ^3^*KD* kidney diseases, ^4^*BND* brain & neurologic disorders, ^5^*RD*respiratory diseases, ^6^*GID* gastrointestinal diseases, ^7^*LD* liver diseases, ^8^*HBD* hematopoietic & blood disorders and ^9^Others: diseases of immunodeficiency, lupus, special diseases, and thyroid.

*Mean Ct (relative viral load): 72 cases a missing in total; 47 cases in the fourth wave; 16 cases in the fifth wave; and 9 cases in the null wave.

### Epidemiological findings

According to Table 1, the daily distribution (based on patients/day) of hospital admissions in the first, second, third, fourth, fifth and sixth waves is 22.5 patients (1598/71), 31.5 patients (2771/88), 25.4 patients (2563/101), 37 patients (2519/68), 32.9 patients (5231/159) and 34 patients (2143/63), respectively.

Furthermore, as shown in Table 1, the daily distribution (based on patients/day) of in-hospital mortality in the first, second, third, fourth, fifth and sixth waves is three deaths (211/71), 2.1 deaths (186/88), 2.3 deaths (234/101), 2.5 deaths (167/68), 0.9 deaths (139/159) and 0.7 deaths (43/63), respectively.

The first wave took place between early March and mid-May 2020. The largest average number of daily admissions and in-hospital mortality was 37 (patients/day) in the fourth wave and the largest average number of daily in-hospital mortality was three (patients/day) in the first wave. The fifth wave started in late June and early July, followed by a gradual increase with a large number of patients admitted in early and late December. The average number of in-hospital mortality and daily admissions was 1 and 33 cases respectively in the fifth wave and 0.7 and 34 cases respectively in the sixth wave.

Across the six waves, the average reported regional incidence increased (4.1, 5.8, 4.6, 6.8, 6 and 6.2 cases per hundred population/day), as did the positivity of diagnostic tests (34.5%, 55.1%, 60.6%, 55.7%, 68.3% and 39%); in-hospital mortality for SARS-CoV-2 positive (0.32, 0.26, 0.28, 0.26, 0.11 and 0.08 deaths per hundred population /day).

### Comorbidities

About one-third of confirmed COVID-19 patients (29.3%) had at least one underlying condition, and the most common comorbidities were CVD (24.8%), diabetes (21.3%) and hypertension (10.1%) (see Table 2 for more details).

### SARS-CoV-2 relative viral load and outcome

According to Table 3, of 11045 rRT-PCR-positive cases for SARS-CoV-2, 75% (8312/11045) have Ct value ≤ 30. Most patients with the highest relative viral load (Ct value of 9-20) were detected in the fifth wave. Further, the rate of ICU admission for patients with a Ct value of 9-20, 21-30 and 31-40 was 5.6% (120/2140), 4.4% (271/6172) and 3.5% (92/2653), respectively. In addition, the rate of in-hospital mortality in patients with a Ct value of 9-20, 21-30 and 31-40 was 8.6% (183/2140), 5.7% (350/6172) and 6.7% (178/2653), respectively.

**Table 3:** Characteristics and outcomes in 11.037 confirmed COVID-19 hospitalized patients in Babol district, according to the Ct value of rRT-PCR from March 7, 2020 to March 20, 2022.

Characteristics	Total	Mean Ct of real-time PCR			*p*-value
		9–20	21–30	31–40	
Overall	10965	2140	6172	2653	–
Median age, yrs, (IQR)	58 (42–68)	56 (40–69)	55 (41–67)	57 (43–69)	0.007
Mean ± SD age, yrs	51±27.3	53.9±20.7	54.4±18	55.2±19.7	0.003
Age group, yrs					
< 18	391 (3.5)	126 (5.9)	143 (2.3)	121 (4.6)	0.020
18–49	3737 (33.8)	678 (31.7)	2234 (36.2)	798 (30.1)	
50–64	3466 (31.4)	645 (30.2)	1964 (31.8)	828 (31.2)	
65–79	2414 (21.9)	470 (22)	1304 (21.1)	627 (23.6)	
≥ 80	1033 (9.4)	220 (10.3)	524 (8.5)	279 (10.9)	
Sex					
Men	5080 (46)	951 (44.4)	2829 (45.8)	1263 (47.6)	0.085
Women	5965 (54)	1189 (55.6)	3343 (54.2)	1390 (52.4)	
Underlying diseases					
CVD^1^	2742 (24.8)	484 (22.6)	1445 (23.4)	791 (29.8)	<0.001
Diabetes	2356 (21.3)	429 (20)	1256 (20.3)	657 (24.8)	<0.001
KD^2^	313 (2.8)	80 (3.7)	132 (2.1)	99 (3.7)	<0.001
Hypertension	1121 (10.1)	194 (9.1)	612 (9.9)	311 (11.7)	0.006
Malignancies	259 (2.3)	57 (2.7)	122 (2)	80 (3)	0.008
BND^3^	463 (4.2)	103 (4.8)	264 (4)	112 (4.2)	0.398
RD^4^	309 (2.8)	53 (2.5)	172 (2.8)	83 (3.1)	0.393
GID^5^	17 (0.2)	2 (0.1)	7 (0.1)	8 (0.3)	0.055
LD^6^	69 (0.6)	12 (0.6)	38 (0.6)	19 (0.7)	0.779
HBD^7^	53 (0.5)	13 (0.6)	25 (0.4)	15 (0.6)	0.398
Pregnancy	136 (1.2)	43 (2)	70 (1.1)	23 (0.9)	0.001
Others^8^	99 (0.9)	17 (0.8)	52 (0.8)	30 (1.1)	0.355
Comorbidities					
No-comorbidities	5757 (52.1)	1140 (53.3)	3375 (54.7)	1198 (45.2)	<0.001
One	3239 (29.3)	629 (29.4)	1711 (27.7)	871 (32.8)	
≥ Two	2049 (18.6)	371 (17.3)	1086 (17.6)	584 (22)	
Hospitalized outcome					
Length of stay, days, median (IQR)	5 (4–8)	5 (4–8)	5 (4–8)	5 (4–8)	0.017
Length of stay, days, Mean **±** SD	7.1±8.1	7.1±7.8	6.9±7.7	7.4±9.3	0.371
ICU admission	485 (4.4)	120 (5.6)	271 (4.4)	92 (3.5)	0.002
In-hospital death	714 (6.5)	183 (8.6)	350 (5.7)	178 (6.7)	<0.001
SARS-CoV-2 infection waves					
First wave	551 (5)	18 (0.8)	226 (3.7)	307 (11.6)	<0.001
Second wave	1527 (13.8)	214 (10)	748 (12.1)	565 (21.3)	
Third wave	1553 (14.1)	112 (5.2)	884 (14.3)	557 (21)	
Fourth wave	1402 (12.7)	444 (20.7)	742 (12)	169 (6.4)	
Fifth wave	3574 (7.6)	919 (42.9)	2207 (35.8)	428 (16.1)	
Sixth wave	836 (7.6)	226 (10.6)	502 (8.1)	108 (4.1)	
Null wave^9^	1602 (14.5)	207 (9.7)	836 (14)	519 (19.6)	

^1^*CVD* cardiovascular diseases, ^2^*KD* kidney diseases, ^3^*BND* brain & neurologic disorders, ^4^*RD* respiratory diseases, ^5^*GID* gastrointestinal diseases, ^6^*LD* liver diseases, ^7^*HBD* hematopoietic & blood disorders, ^8^Others: including diseases of immunodeficiency, lupus, special diseases, and thyroid, ^9^Null Wave: related to between waves of SARS-CoV-2 Infections (about 208 days).

### Clinical outcomes

Of the 24,287 hospitalized patients with suspected COVID-19, 1236 died (5.1%), including 682 (55.2%) male patients. Among 11,045 confirmed COVID-19 patients, 714 deaths (6.5%) were recorded, including 380 (53.2%) males and 334 (46.8%) females. The deceased cases had a mean age of 66.1 ± 16.7 years compared to those who were discharged (53.7 ± 18.8) (Tables 2 and 4).

**Table 4:** Clinical and demographic characteristics for 23,051 hospitalized COVID-19 patients in Babol district, among survivors and non-survivors and rRT-PCR positive cases from March 7, 2020 to March 20, 2022.

Variable	Total			rRT-PCR Positive		
	Survivors (N= 23051), n (%)	No survivors (N= 1236), n (%)	*P* value	Survivors (N= 10331), n (%)	No survivors (N= 714), n (%)	*P* value
Median age, yrs, (IQR)	56 (38–70)	69 (55–80)	<0.001	55 (41–67)	68 (55–79)	<0.001
Mean ± SD age, yrs	51.9±23.9	65.8±18.2	<0.001	53.7±18.8	66.12±16.7	<0.001
Age group, yrs						
< 18	2708 (11.8)	23 (1.9)	<0.001	384 (3.7)	7 (1)	<0.001
18–49	6356 (27.7)	191 (15.5)		3629 (35.1)	108 (15.1)	
50–64	6058 (26.4)	301 (24.5)		3273 (31.1)	193 (27)	
65–79	5257 (22.9)	387 (31.4)		2184 (21.1)	230 (32.2)	
≥ 80	2589 (11.3)	329 (26.7)		857 (8.3)	176 (24.6)	
Sex						
Male	11,242 (48.8)	682 (55.2)	<0.001	4700 (45.5)	380 (53.2)	<0.001
Female	11,809 (51.2)	554 (44.8)		5631 (54.5)	334 (46.8)	
Comorbidity						
CVD^1^	6321 (27.4)	461 (37.3)	<0.001	2488 (24.1)	254 (35.6)	<0.001
Diabetes	4657 (20.2)	353 (28.6)	<0.001	2137 (20.7)	219 (30.7)	<0.001
KD^2^	942 (4.1)	84 (6.8)	<0.001	268 (2.6)	44 (6.2)	<0.001
Hypertension	2414 (10.5)	128 (10.4)	0.896	1050 (10.2)	71 (9.9)	0.851
Malignancies	1233 (5.3)	80 (6.5)	0.089	231 (2.2)	28 (3.9)	0.004
BND^3^	1359 (5.9)	94 (7.6)	0.014	419 (4.1)	44 (6.2)	0.007
RD^4^	783 (3.4)	78 (6.3)	<0.001	268 (2.6)	41 (5.7)	<0.001
GID^5^	93 (0.4)	7 (0.6)	0.384	16 (0.2)	1 (0.1)	0.922
LD^6^	188 (0.8)	16 (1.3)	0.072	62 (0.6)	7 (1)	0.212
HBD^7^	190 (0.8)	11 (0.9)	0.804	49 (0.5)	4 (0.6)	0.748
Pregnancy	280 (1.2)	1 (0.1)	<0.001	135 (1.3)	1 (0.1)	0.006
Others^8^	220 (1)	11 (0.9)	0.820	93 (0.9)	6 (0.8)	0.870
Comorbidity						
No-comorbidity	10,829 (47)	380 (30.7)	<0.001	5512 (53.4)	245 (34.3)	<0.001
One	7547 (32.7)	514 (41.6)		2959 (28.6)	280 (39.2)	
Two ≤	4675 (20.3)	342 (27.7)		1860 (18)	189 (26.5)	
SARS-CoV-2 waves						
First	1378 (6)	211 (17.1)	<0.001	425 (4.1)	126 (17.6)	<0.001
Second	2585 (11.2)	186 (15)		1402 (13.6)	124 (7.4)	
Third	2329 (10.1)	234 (18.9)		1398 (13.5)	154 (21.6)	
Fourth	2352 (10.2)	167 (13.5)		1307 (12.7)	95 (13.3)	
Fifth	5092 (22.1)	139 (11.2)		3477 (33.7)	96 (13.4)	
Sixth	2100 (9.1)	43 (3.5)		806 (7.8)	29 (14.1)	
Null wave^9^	7206 (31.3)	256 (20.7)		1508 (14.6)	90 (12.6)	
Hospitalized outcome						
Length of stay, days, median (IQR)	5 (4–8)	7 (3–15)	<0.001	5 (4–7)	7 (4–14)	<0.001
Length of stay, days, mean **±** SD	7.5±8.9	11.7±14.3	<0.001	6.8±7.6	11.2±12.4	<0.001

^1^*CVD* cardiovascular diseases, ^2^*KD* kidney diseases, ^3^*BND* brain & neurologic disorders, ^4^*RD* respiratory diseases, ^5^*GID* gastrointestinal diseases, ^6^*LD* liver diseases, ^7^*HBD* hematopoietic & blood disorders, ^8^Others: including diseases of immunodeficiency, lupus, special diseases, and thyroid, ^9^Null Wave: related to between waves of SARS-CoV-2 infections (about 208 days).

In-hospital mortality was 1%, 15.1%, 27%, 32.2%, and 24.6% in the age groups <18 years, 18–49 years, 50–64 years, 65–79 years and ≥ 85 years, respectively. In addition, the ICU admission rate was 3.7% (891 patients) and 4.4% (485 patients) for patients with suspected and confirmed COVID-19. Further findings are listed in Tables 2, 3 and 4. Besides, by hospital indication, more than 68% (7594 of 11045) of patients admitted with a positive rRT-PCR diagnostic test were age <65 years, but the prevalence of death was significantly higher in those ≥ 65 (56.9%) years of age (P<0.001, Tables 3 and 4). Additionally, more women (54%; 5965/11045) were hospitalized with a positive rRT-PCR test, while more men died (men: 7.4%; 380/5080 vs. women: 5.6%; 334/5965) (P < 0.001).

The frequency distribution of mortality among COVID-19 patients in the different waves was as follows: 126 cases (17.6%), 124 cases (17.4%), 154 cases (21.6%), 95 cases (13.3%), 96 cases (13.4%), 29 cases (4.1%), and 90 (12.6%) in the first, second, third, fourth, fifth, sixth and between waves, respectively.

Of 11,037 confirmed hospitalized patients, the lowest COVID-19 rate was in the first wave at 5% (551 patients), while the highest rate was in the fifth wave at 32.4% (3573 patients) (Table 3).

The median and mean duration of hospitalization for discharged COVID-19 patients was 5 (IQR: 4-8) and 6.8 (SD: 7.6) days, respectively. For deceased COVID-19 patients, the median and mean duration of hospitalization was 7 (IQR: 3-15) and 11.2 (SD: 12.4) days, respectively (Table 4).

Furthermore, the median and mean duration of hospitalization for discharged COVID-19 patients in the first, second, third, fourth, fifth, and sixth waves was 6 (IQR: 4-10) and 7.7 (SD: 7.2), 6 (IQR: 3-9) and 8.2 (SD: 9.8), 5 (IQR: 4-8) and 6.9 (SD: 6.3), 5 (IQR: 4-7) and 6.6 (SD: 9.4), 5 (IQR: 4-7) and 6.7 (SD: 7.5), 5 (IQR: 3-8) and 6.3 (SD: 5.7) days, respectively (Table 2).

### Survival analysis

Cox regression was used to assess the factors influencing patient survival. In the first step, all variables including age, gender, rRT-PCR result, mean Ct value, underlying diseases and different waves were entered into the model. The coefficients were estimated using the stepwise backward method (likelihood ratio). In step eight, the variables age, gender, rRT-PCR result, mean Ct value, KD, hypertension, malignant disease, respiratory disease (RD) and liver as underlying disease as well as waves remained in the model.

**Figure 2:**
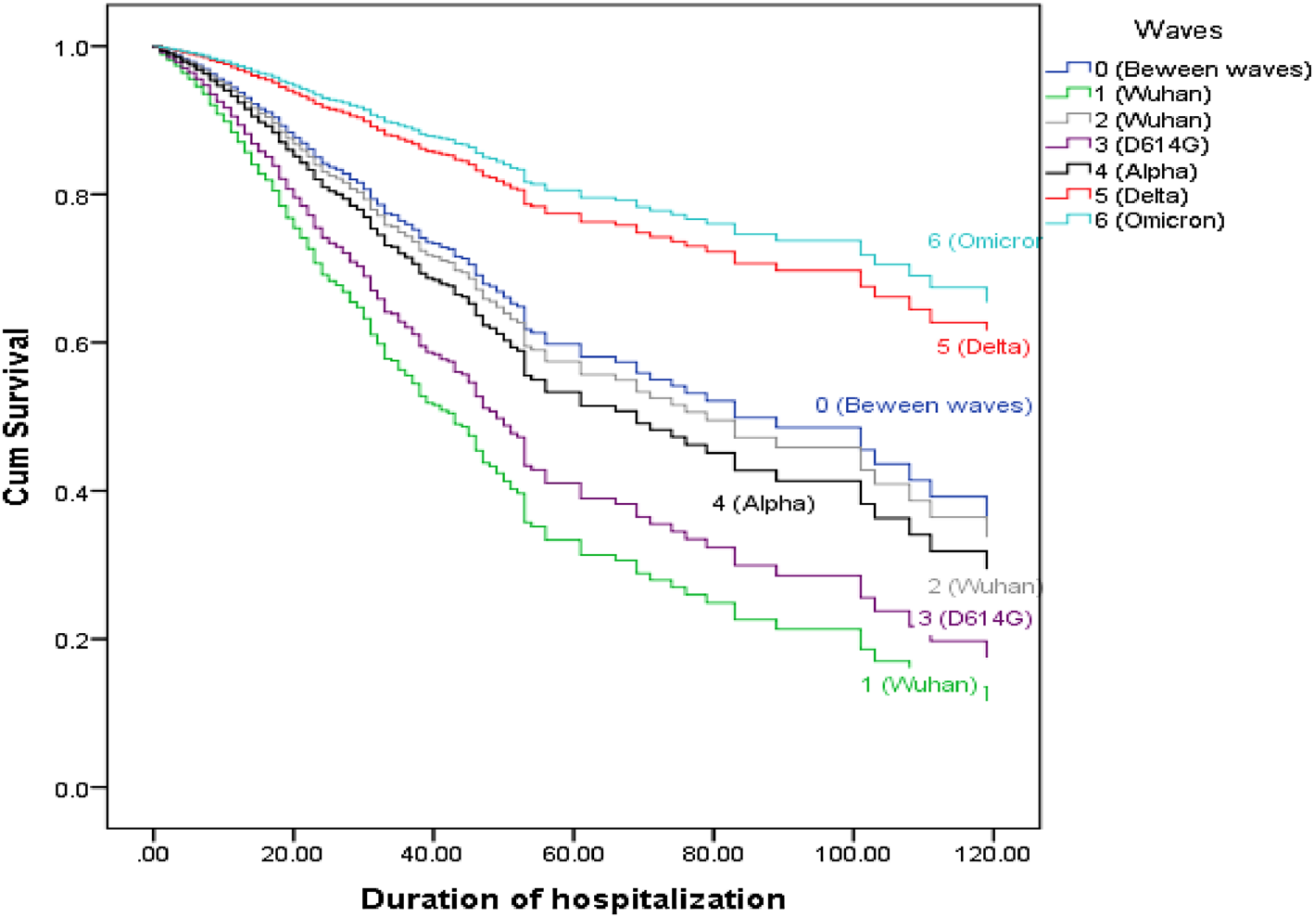
Survival (length of hospitalization to discharge alive or mortality) of COVID-19 patients between the different waves.

Table 5 illustrates that the mortality risk for positive rRT-PCR cases is 0.38 higher than for negative cases (HR = 1.38, 95% CI 1.16–1.64). Moreover, the risk of mortality was higher in patients with mean Ct: 9–20 than in patients with mean Ct: 31–40 (HR = 1.93, 95% CI 1.56–2.39), which is almost twice as high as in patients with mean Ct: 31–40 and negative rRT-PCR. The risk of death therefore also increases with increasing viral load. Furthermore, the fifth and sixth waves had a lower mortality risk compared to the null waves and seemed to have protected against death (like hypertension, which protected against 30% of the risk). As can be seen in Fig. 2, patient survival (length of hospitalization to discharge alive or dying) was significantly higher in the fifth and sixth waves (Delta and Omicron) than in the null wave, and survival in the first wave (Wuhan) was significantly lower (Fig. 2).

**Table 5:** Survival analysis of COVID-19 hospitalized patients in Babol district, Northern Iran, from March 7, 2020 to March 20, 2022.

Parameter	Hazard ratio	95% CI HR	P-value
Age	1.03	1.02–1.03	<0.001
Sex (male)^1^	0.85	0.76–0.95	0.004
Real-time PCR positive	1.38	1.16–1.64	<0.001
Mean Ct^2^			
9–20	1.93	1.56–2.39	<0.001
21–30	1.19	0.99–1.43	0.058
Underlying diseases			
Kidney diseases	1.26	1.01–1.58	0.040
Hypertension	0.669	0.56–0.81	<0.001
Malignancies	1.36	1.08–1.72	0.008
Respiratory diseases	1.34	1.06–1.68	0.013
Liver diseases	1.65	1.00–2.71	0.049
Disease waves			
1	2.13	1.77–2.57	<0.001
2	1.08	0.89–1.31	0.443
3	1.73	1.44–2.08	<0.001
4	1.22	1.00–1.49	0.051
5	0.5	0.40–0.62	<0.001
6	0.42	0.30–0.58	<0.001

^1^Reference category: female, ^2^Reference category: Real-time PCR negative & mean Ct (31–40).

## Discussion

This study investigated the demographic, epidemiological and rRT-PCR findings and outcomes of hospitalized COVID-19 patients in Babol district of northern Iran during the two years of the pandemic. In the current study, 24,287 patients with suspected COVID-19 and 11,045 patients with confirmed SARS-CoV-2 infections were admitted to the hospitals. CVD, diabetes, BND, cancer, KD, RD, pregnancy, age, gender and pandemic waves with risk factor(s) were associated with COVID-19 mortality.

The in-hospital mortality rate varied in previous reports from Iran: 8.5% in a cohort from Ardabil province^17^, 9.7% in a cohort from Kermanshah province^18^, 9.8% in a cohort from Yazd province^19^, about 10% in a cohort from Tehran province^20^, and 27.62% in Mashhad^21^. The in-hospital mortality rate differed from previous reports from Iran, which reported 8.5%, 9.7%, 9.8%, 10% and 27.6% in a cohort of Ardabil, Kermanshah, Yazd, Tehran and Mashhad, respectively^17–21^.

Despite the fact that the total number of hospitalized cases and positive SARS-CoV-2 cases in the fifth wave (relative to the Delta wave) (21.5% and 32.4%, respectively) was more than twice as high as in the first (6.6% and 5%), second (11.4% and 13.8%), third (10.6% and 14.1%) fourth (10.4% and 12.7%), and sixth (related to Omicron) waves (8.8% and 7.6%), the in-hospital mortality rate in the first (related to Wuhan) wave (13.2% and 22.9%) was almost three times as high as in the second (6.7% and 8.1%), third (9.1% and 9.9%), fourth (6.6% and 6.8%), fifth (2.7% and 2.7%) and sixth waves (2% and 3.5%).

Therefore, a decreasing trend in the mortality rate of patients was observed over the course of the study, while the highest mortality rate was recorded in the first wave of SARS-CoV-2. In the current study, 4.4% of patients with rRT-PCR confirmed COVID-19 were admitted to the ICU and in-hospital mortality was 6.5%. In a national retrospective cohort study in Iran between February and April 2020, in-hospital mortality within 30 days was 24.4% (5693 of 23,367 patients)^20^. In a study in the province of Yazd, central Iran, the mortality rate was 9.8% (2185/ 24,563)^19^. In a nationwide study of Brazilian hospitalized patients with rRT-PCR confirmed COVID-19, 59% (4002 of 79,687) of patients were admitted to the ICU and in-hospital mortality was 38% (87,515 of 232,036 patients)^22^. The in-hospital mortality rate for COVID-19 patients in India^23^, Oman^24^, and the USA^25^ was 13.7%, 21.4%, and 26%, respectively. Although hospital admission conditions, patient characteristics, case descriptions, etc. differ from country to country, the mortality of COVID-19 patients could be influenced by differences between demand, capacity and the lack of skilled ICU staff.

Similar to the Spanish reports by Iftimie et al.^26^ 22.9% of hospitalized confirmed COVID-19 patients died in the first wave, and this percentage was reduced to 2.7% and 3.5% in the fifth and sixth waves, respectively. The in-hospital mortality rate in five waves of COVID‑19 in Mexico was 45.1%^27^.

In the current study, the in-hospital mortality rate associated with COVID-19 was lowest in the age group 0–17 years and increased in patients 65≥ years. Some studies have shown that advancing age is associated with a weakened immune system and an increased risk of comorbidities, leading to higher COVID mortality^28^. In the ongoing study, more than half of the hospitalized cases were male patients, which is consistent with other studies^18,29,30^. Age is the most important risk factor for severe COVID-19 outcomes. Patients with one or more underlying diseases are also at high risk^31–33^. Furthermore, the Italian study found that age and being male are separate factors that increase the risk of mortality from COVID-19, regardless of whether the patient was hospitalized or not^34^. Additionally, hospitalization during the second and third waves was linked to a lower risk of death from COVID-19 compared to the first wave. However, there was no significant difference in survival rates for patients over the age of 75^35^. In line with previous studies, the most common underlying diseases were CVD, diabetes, and hypertension^36,37^. The results of the current study suggested the association between several underlying diseases, including CVD, diabetes, BND, KD, cancer, pregnancy, and RD, and COVID-19-related mortality, which is similar to the findings of Hesni et al.^18^, Namayandeh et al.^19^, Singhal et al.^38^, Thakur et al.^39^, Gu et al.^37^, Flaherty et al.^36^ and Iftimie et al.^26^. In the present study, diabetes was recognized as the second major underlying disease associated with COVID-19 mortality, which is in line with the finding of Iftimie et al.^26^.

Interestingly, the in-hospital mortality rate of COVID-19 in pregnant women in the present study was very low (1 case per 281 pregnant women). Similarly, a multicenter retrospective cohort study of COVID-19 in hospitalized pregnant women from Kermanshah province in Iran evaluated one case per 259 pregnant women^18^ and a systematic review reported that the mortality of pregnant women with COVID-19 was lower than that of COVID-19 patients overall^40^. A study of pregnant women in Mexico also found no difference in perinatal outcomes between SARS-CoV-2 positive and negative cases^41^. This may be related to the fact that younger pregnant women may have a lower risk of underlying diseases.

A multicentre cohort study on COVID-19 in hospitalized pregnant women from the USA reported 44 (69%) pregnant women with severe disease and 20 (31%) with critical illness^42^.

Although the number of confirmed COVID-19 patients was lowest in the first wave (5%; 551/11037), the highest rate was recorded in the fifth wave (32.4%; 3573/11037). Furthermore, the in-hospital mortality rate in the first wave was 22.9% (126/551) and thus significantly higher than in the fifth wave (2.9%).

Babol district experienced six major waves of hospitalizations and deaths in the summer and fall compared to other provinces. In contrast to other reports from Iranian health authorities, the results of the present study also represented an increase in the total number of hospitalizations during the fifth wave, but in deaths during the first wave compared to the subsequent and later waves^11^. It is worth noting that the fifth wave was accompanied by the emergence of Delta variants of SARS-CoV-2 in our region, which are more transmissible than the first wave associated with the ancestral Wuhan strain. It may be related to the start of mass vaccination against COVID-19 on February 21, 2021 and the provision of appropriate equipment, increase in resource capacity, more appropriate treatment and more qualified treatment, trained ICU staff, and containment measures implemented during the pandemic.

The strengths of the present study were the large sample size with suspected and confirmed suspected COVID-19 patients; therefore, it can provide a comprehensive picture of hospitalized patients with COVID-19. A positive aspect of this investigation was the large sample size, which included confirmed, probable, and suspected cases of COVID-19. Hence, it has the potential to provide a comprehensive overview of individuals hospitalized with COVID-19 infection. Our study encountered several limitations. First, certain variables, such as hospitalized treatments and laboratory results, were not disclosed, which limited our ability to draw conclusions about mortality based on the available data. Additionally, the data on underlying diseases and comorbidities were based on self-reporting, which could not be verified for accuracy. Thirdly, as with typical retrospective studies, there may be an unintentional bias in patient selection. This study focused exclusively on patients admitted to hospitals and drew its data from four specific healthcare facilities. To achieve broader generalizability, it is essential to repeat these results in different hospitals. Consequently, the results of the current study may not fully represent the comprehensive landscape of COVID-19 in Iran. Fourth, the present study was unable to assess the direct impact of the vaccine on disease severity as there was no information on the vaccination status of the patients. In particular, vaccination has been reported to reduce the risk of hospitalization and the development of severe symptoms. This factor may elucidate variations in outcomes observed during the different COVID-19 waves after the general introduction of vaccination in Iran.

## Conclusions

The various clinical characteristics and outcomes of hospitalized patients with COVID-19 in each wave, up to the sixth wave in Iran, were identified. Overall, the in-hospital mortality rate was 6.5%, with male gender, advanced age, and comorbidities significantly increasing the mortality rate. Additionally, the results of the ongoing study revealed an increase in the total number of hospitalizations and confirmed SARS-CoV-2 cases until the fifth wave of the pandemic in Iran. It is noteworthy that the patients of the fifth COVID-19 wave were significantly younger than those of the other waves and that the mortality rate was the lowest. The confirmed COVID-19 patients of the fifth wave had either no or fewer comorbidities, and the age of the patients who died was higher than that of the survivors. The impact of these findings could help ensure that the Iranian healthcare system is prepared for future waves of COVID-19. However, it is imperative that the health authorities address this revelation immediately and ensure improved medical care and the implementation of preventive measures for health facilities located outside the central locations.

## Data Availability

The data are available on request from the corresponding author.
